# Systematic assessment of pharmaceutical prescriptions in association with cancer risk: a method to conduct a population-wide medication-wide longitudinal study

**DOI:** 10.1038/srep31308

**Published:** 2016-08-10

**Authors:** Chirag J. Patel, Jianguang Ji, Jan Sundquist, John P. A. Ioannidis, Kristina Sundquist

**Affiliations:** 1 Department of Biomedical Informatics, Harvard Medical School, 10 Shattuck St, Boston, MA, 02115 USA; 2 Center for Primary Health Care Research, Department of Clinical Sciences, Lund University, Clinical Research Centre (CRC), building 28, floor 11, Jan Waldenströms gata 35, Skåne University Hospital, SE-205 02 Malmö, Sweden; 3 Stanford Prevention Research Center, Department of Medicine, and Department of Health Research and Policy, Stanford University School of Medicine, 1265 Welch Road, Stanford, CA, 94305, USA

## Abstract

It is a public health priority to identify the adverse and non-adverse associations between pharmaceutical medications and cancer. We search for and evaluate associations between all prescribed medications and longitudinal cancer risk in participants of the Swedish Cancer Register (N = 9,014,975). We associated 552 different medications with incident cancer risk (any, breast, colon, and prostate) during 5.5 years of follow-up (7/1/2005-12/31/2010) in two types of statistical models, time-to-event and case-crossover. After multiple hypotheses correction and replication, 141 (26%) drugs were associated with any cancer in a time-to-event analysis constraining drug exposure to 1 year before first cancer diagnosis and adjusting for history of medication use. In a case-crossover analysis, 36 drugs (7%) were associated with decreased cancer risk. 12 drugs were found in common in both analyses with concordant direction of association. We found 14, 10, 7% of all drugs associated with colon, prostate, and breast cancers in time-to-event models. We only found 1, 2%, and 0% for these cancers, respectively, in case-crossover analyses. Pharmacoepidemiologic analyses of cancer risk are sensitive to modeling choices and false-positive findings are a threat. Medication-wide analyses using different analytical models may help suggest consistent signals of increased cancer risk.

Identification of associations between commonly prescribed drugs and major adverse events, especially cancer risk is a priority in pharmacoepidemiology. During the pre-approval process a drug is usually tested in randomized trials, thought to be the standard in avoiding biases found in non-randomized observational studies. It is very difficult to characterize some possible long-term effects of drugs on chronic outcomes, such as cancer, in these trials because of limited follow-up, small sample size, limited types of populations examined, fixed regimens, and suboptimal data collection and reporting of such outcomes in the randomized trial setting^1^ Thus, cancer risk assessment is usually performed in large observational datasets. However, for most of pharmacoepidemiological links to cancer, there has been controversy about their validity. Furthermore, hypotheses in pharmacoepidemiological studies often are concerned with one drug or a few drugs at a time on one or a few selected (or selectively reported) outcome(s) and analyses thereof, and this may lead to irreproducible and biased effects^2^,^3^.

The availability of large-scale databases and the evolution of computational methods currently allows testing for associations between prescriptions and long-term outcomes in a systematic manner, where a large number of drugs can be tested for association with adverse events in the same analysis^4^,^5^,^6^. Such approaches may not necessarily correct for confounding or other biases, but in theory they have the ability to avoid selective analysis and outcome reporting and they can also adjust their results for the multiplicity of the analyses performed. We have recently developed methods for environment-wide association studies (EWASs), aiming to search for and validate environmental factors associated with disease and disease-related phenotypes testing multiple exposures at the same time within the same analysis^7^,^8^,^9^,^10^,^11^,^12^. Recently, Ryan and colleagues performed a medication-wide association study and scanned 88 to 118 drugs in a case-control setting in cohorts derived from large claims and electronic health record databases focusing on associations for four acute outcomes (including myocardial infarction, acute liver failure, acute renal failure, and upper gastrointestinal bleeding)^5^.

Here, we extend these methods to systematically evaluate associations of all prescribed drugs (N = 552) with longitudinal cancer risk in the Swedish population from 2005 to 2010 (N = 9,014,975), leveraging a longitudinal linkage between the Swedish Prescribed Drug Register and the Cancer Register. These registries have excellent coverage of the Swedish population, with <1% missing information. In a secondary analysis, we aimed to search for drugs putatively associated with any, breast, colon, and prostate future cancer risk. We explored different analytical approaches, so as to understand whether the results are sensitive to the choice of analysis and assumptions made. Drug associations that tend to give inconsistent results with different models and assumptions may be spurious, while those with consistent signals may be worth considering further. Second, we investigate the ease at which inferences may be made when conducting large-scale associations in large sample sizes that represent an entire nation. We finally compared the results of this approach against proposed associations for increased cancer risk that have been reported in meta-analyses of diverse medications in the published literature^13^.

## Methods

**Figure 1:**
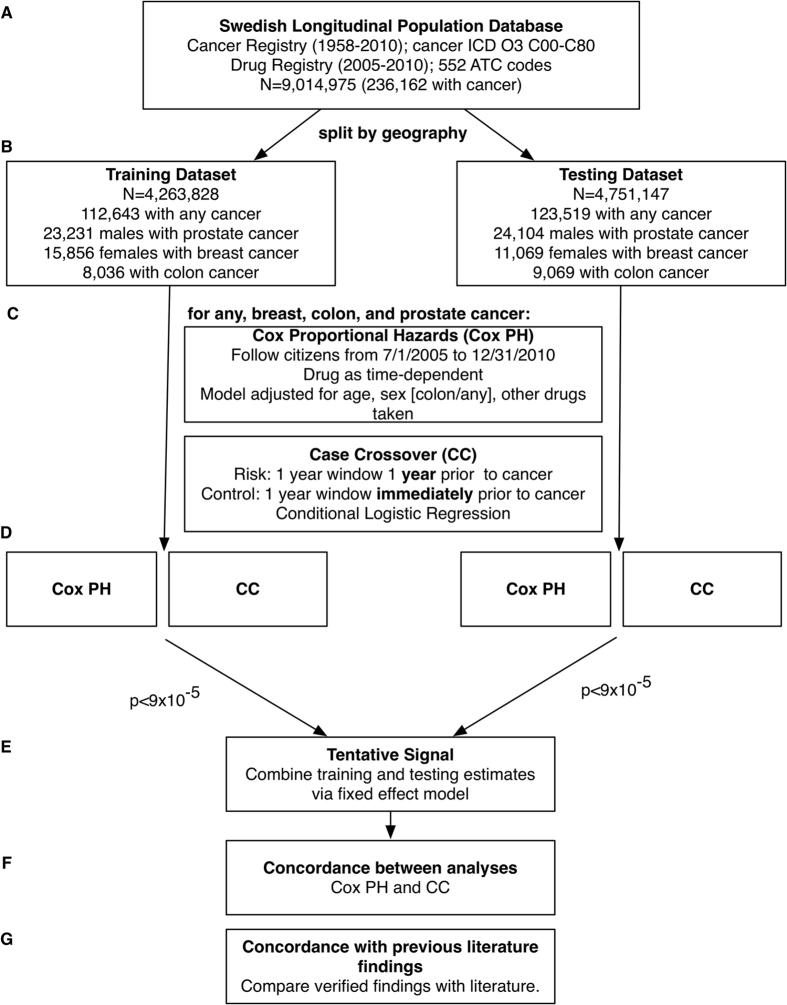
Overview of method to associate 552 medications with cancer risk. (**A**) The data source was the Swedish longitudinal database, consisting of the Cancer Registry and Prescribed Drug Register (“ATC” = Anatomical Therapeutic Classification). (**B**) We split data into a training and testing dataset by location. (**C**) We conducted 2 possible analyses for each cancer type (e.g., any, breast, colon, and prostate) with (**B**), Cox proportional hazards regression (Cox PH), and a Case-Crossover (CC) analyses, only adjusting for sex in the any cancer or colon cancer outcomes, (**D**) Association testing. (**E**) Claiming a verified signal (p < 10^−5^ in both training and testing datasets) (**F**) Estimate concordance between each analysis, and (**G**) Estimate concordance between each analysis and the previous literature.

We conducted a prescription-wide association study on cancer risk for people living in Sweden with median age of 38 years (interquartile range 20 to 57), utilizing a longitudinal research cohort database maintained by the Center for Primary Health Care Research at Lund University in Malmö, Sweden (Fig. 1).

We confirm that the all experimental protocols were approved by Regional Ethics Committee of Lund University in Sweden. Second, our methods are in accordance with the European Network of Centres for Pharmacoepidemiology and Pharmacovigiliance (ENCEPP) guidelines^14^ and Strengthening the reporting of observational studies in epidemiology (STROBE).

We assembled the database by merging the Swedish Cancer Register, which contains date of cancer diagnosis of all Swedish residents from years 1958–2010, and the Prescribed Drug Register, which contains 552 unique types of drug prescriptions dispensed to Swedish residents^15^ from July 1^st^, 2005 to December 31^st^, 2010 (Fig. 1A). The registry contains greater than 99% of all prescriptions dispensed in Sweden (less than 1% are missing). In Sweden, it is compulsory for health care providers to report all detected cancer cases to the cancer registry. Along with these cases, a report is sent describing clinical, morphological, and/or autopsy results used to diagnose cancers. There are six regional registries associated with the national oncological centers in Sweden where the coding is performed. The regionalization implies a close contact between the registry and the reporting physician, simplifying the task of correcting and checking the diagnoses of cancer cases.

The outcome of interest was time to first diagnosis of any, breast, colon, or prostate cancer as determined by International Classification of Diseases “O3” billing codes. For any cancer, we utilized codes C00-C80 (all codes in this range). For breast cancer (in females) we utilized codes C50 (C50.0-C50.9). For prostate cancer in males, we utilized code C61. For colon cancer, we utilized codes C18(C18.0-C18.9). We analyzed individuals starting when nation-wide drug surveillance began on July 1^st^, 2005, to the end of follow-up in December 31^st^, 2010 (total of 2009 days). There were a grand total of 9,014,975 individuals eligible for analysis in this time frame (236,162 individuals with a billing code for any cancer). We divided the population into two datasets randomly by geography (based on the 21 counties that comprise the entire country), assigning individuals into either a “training” set (N = 4,263,828; 112,643 any cancer cases) and a “testing” set (N = 4,751,147; 123,519 any cancer cases). The training set consisted of individuals from the largest urban area and ten smaller urban or rural areas, while the testing set consisted of individuals from two other large urban areas and eight smaller urban or rural areas. Assignment of geographical region to the training or testing sets was done at random. We used the training dataset to scan for prescriptions associated with cancer risk and the testing dataset to verify associations (Fig. 1B). See Appendix (Table S1) for sample sizes by year of surveillance, training/testing dataset, and cancer type.

The Sweden Prescribed Drug Register contains information on prescriptions on 552 different types of drugs as classified by the Anatomical Therapeutic Chemical (ATC) classification system maintained by the World Health Organization^16^. Briefly, there are four levels of ATC classification: the first level is the anatomical main group for the drug, 2^nd^ is the therapeutic subgroup, 3^rd^ is the pharmacological subgroup, 4^th^ is the chemical subgroup, and 5^th^ is the chemical substance. The Sweden Prescribed Drug Register contains the date an individual received their prescription up to the 4^th^ ATC level classification (chemical subgroup).

### Systematic scan of prescriptions associated with time to cancer

We associated each of the 552 possible drug prescriptions with (1) any, (2) breast, (3) prostate, and (4) colon cancer risk using two different modeling strategies in order to see how much the results are affected by the model choice and its assumptions.

First, we performed Cox proportional time-to-event regressions in the training and testing datasets in (1) any, (2) breast, (3) prostate, and (4) colon cancers separately. Each prescription was analyzed as a time-dependent variable, so that an individual is considered “non-exposed” to a drug before the date of prescription and “exposed” after the date of prescription. We censored individuals at the time of diagnosis of first cancer or at the end of the study period (December 31, 2010), whichever occurred first (Fig. 1C,D). Furthermore, we only considered a patient “exposed” to a drug if and only if the date of prescription came 180 days after the start of surveillance (December 28, 2005 for the July 1^st^, 2005 start date). We chose to impose a 180-day window between the start of surveillance and exposure period to mitigate the chances of prevalent exposure (exposure coming before the surveillance period). Specifically, patients were considered to be “exposed” (1) 1 year after the prescription date and (2) if the date of prescription occurred 180 days (6 months) after the start of surveillance. In other words, patients are considered “exposed” 1 year after the prescription date and the prescription must have occurred 180 days after the start of surveillance.

For any and colon cancer, we adjusted all Cox models by age (as a time-dependent variable), sex, and prescription of any other drugs (coded as 0/1 if not on any other drug/on any other drug) prescribed at anytime during the surveillance period. For breast and prostate cancer, we only considered females and males respectively and did not adjust for sex. Thus, the model was specified to associate the *i*th drug (out of 552 total drugs) for any cancer was:


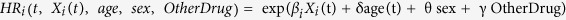


where *X*_*i*_(*t*) is 0 where t is less than 180 days after the start of surveillance or if t is greater than the end of surveillance. *X*_*i*_(*t*) is 1 in non-cancer patients if *t* is greater than 180 days of surveillance. *X*_*i*_(*t*) is 1 in cancer patients if t is greater than 180 days and t is greater than 365 days (1 year) before cancer diagnosis. OtherDrug is 1 if a patient is on another drug during the surveillance time other than *X*_*i*_ and is 0 if the patient has not been prescribed any other drugs. Hazard Ratios (HR, or exp (*β*_*i*_)) describe effect sizes for Cox analyses for a drug *i*. Specifically, HR reflect the change in risk for cancer in exposed versus unexposed individuals. Number of days was the underlying timescale and patients were censored either at the time of first cancer, emigration, or at the end of the surveillance window.

We modeled site-specific cancers in a similar way. To associate drugs with colon cancer risk, we eliminating breast, prostate, and other cancers from the cohort. To associate drugs with female breast cancer, we eliminated colon, prostate, and other cancers; however, we did not include sex as an adjustment variable. Finally, to associate drugs with prostate cancer risk, we eliminated cases with colon, breast, and other cancers. We did not include sex as an adjustment variable in these models.

We used Bonferroni-corrected p-values to adjust for multiple comparisons. A p-value of 9 × 10^−5^ (0.05/552) was considered significant and a finding was considered as a “tentative signal” if it achieved Bonferroni-level of significance in both training and testing datasets and had consistent log(HR) across datasets (had the same sign, either both negative or both positive). We computed an overall effect size and p-value combining training/test datasets with a fixed effect meta-analytic technique. We acknowledge there are less conservative ways to adjust for multiple tests, such as the False Discovery Rate (FDR)^17^. Since our analyses pertained to a large registry sample comprising of the entire population of Sweden, we had more than adequate power to detect prescription drugs at the Bonferroni-level of significance (9 × 10^−5^). Specifically, to detect a Cox proportional hazard ratio of 1.1 (10% increased relative risk for cancer) with sample sizes of 4.5 M, and for a drug with 1/200 patients, we computed 95% power for detecting a result with p-value of 9 × 10^−5^.

Unless stated, all effects are the overall effect size and p-value (Fig. 1E). We visualized findings using “Manhattan plots” and “Volcano plots”. Manhattan plots display the −log10 transformed p-value (on the y-axis) for each drug (x-axis). P-values are −log10 transformed to highlight lower p-values that appear higher on the plot. Volcano plots describe simultaneously the joint distribution of p-values and effect sizes through a plot of −log10 of the p-value versus the association size for each drug.

The second analytical approach used a case-crossover analysis^18^ to mitigate the possibility of time-invariant confounding bias among only cases (patients who are diagnosed with cancer) and to estimate the differences in estimates under a different analysis scenario. While a case-crossover design is typically used to investigate acute outcomes^19^, here we modified the method to investigate an outcome such as cancer diagnosis. Specifically, an “at-risk” and “control” period is determined for each cancer case and therefore each individual serves as his/her control. In this investigation, the “at-risk” period was a 1-year window that spanned the period of 2 years before cancer diagnosis to 1 year before cancer diagnosis. This window had to be at least 180 days after the start of surveillance. If a prescription date for a drug occurred in the “at-risk” period, the patient was “exposed”. The “control” period was the 1-year window just prior to cancer diagnosis. Therefore, if the drug occurred in a 1-year window just prior to the date of cancer diagnosis, the patient was considered to be “unexposed”. The time window is thus the same as what we considered for the Cox model. Individuals that were prescribed drugs after the cancer date were not considered.

Like in the Cox models, we adjusted all case-crossover models by prescription of any other drugs prescribed during the at-risk and/or the control period (coded as 0/1 if not on any other drug/on any other drug). We estimated the association between drugs prescribed in the at-risk period versus control period in the case-crossover analysis by using a conditional logistic regression model. Each “stratum” in a conditional logistic model in a case-crossover model corresponds to an individual. In the case-crossover analysis, Odds Ratios (OR, or exp(*β*_*i*_)) describe effect sizes for the *i*th drug. Specifically, OR reflect the odds for cancer for individuals exposed to a drug in the at-risk versus control time periods defined a priori. We executed a case-crossover for each of the 552 drugs in (1) any, (2) breast, (3) prostate, and (4) colon separately.

Similarly to the Cox analysis above, we used Bonferroni-corrected p-values to adjust for multiple comparisons. A p-value of 9 × 10^−5^ (0.05/552) was considered significant and a finding was considered as a “tentative signal” if it achieved Bonferroni-level of significance in both training and testing datasets and had consistent log(OR) across datasets (had the same sign, either both negative or both positive). We computed an overall effect size and p-value combining training/test datasets with a fixed-effect meta-analytic technique. Unless stated, all effects are the overall effect size and p-value (Fig. 1E).

### Agreement between prescription-wide findings with previously reported findings

Several commonly used medications have been associated with higher cancer risk (HRs >1 or odds ratios [OR] >1) in at least one previously published meta-analysis. Specifically, we used a previous umbrella review by one of us (JPAI) that had systematically assembled a list of 9 unique drugs or drug combinations from 14 meta-analytic studies that had reported a statistically significant increase risk for cancer (as documented in ref. 13, and excluding sex hormones, and malignancies occurring after immunosuppression for transplantation or after treatment of another malignancy). Each of these meta-analytic studies combined evidence from 2 or more individual investigations examining the association between a drug and a cancer outcome (e.g., risk for all cancer types combined or some specific cancer type). The drugs in this list included medications used for control and prevention of chronic disease, such as hyperglycemia and heart disease including thiazolidinedione, insulin, ACE (angiotensin converting enzyme) inhibitors, angiotensin II blockers (ARBs), and diuretics. They also included tumor necrosis factor inhibitors (TNFi), methotrexate, cyclophosphamide, and prednisolone. As reviewed in ref. 13, other studies or meta-analyses on the same medication may not have found an increased risk, but at least one meta-analysis had found such an increased cancer risk. We observed the agreement between our prescription-wide analyses and these previously published claims for increased cancer risk.

### Category-level associations in any cancer

We exploited the ATC hierarchy to estimate category-level associations (e.g., A01 is a category that represents drugs for “Stomatological Preparations”) in any cancer. Briefly, we associated exposure to any of the drugs in a given category (e.g., exposure to A01 is exposure to any of A01AA, A01AB, A01AC, and A01AD) to estimate the difference between the category-level association and drug-level associations. We hypothesized that associations that were robust to confounding by indications would be different in their association size with respect to the category-level association or other drugs used for the same indications.

All analyses were conducted with SAS and R statistical analysis software^20^.

## Results

The average age for individuals in the training and testing dataset was 39.6 (median = 39, lower quartile = 20, upper quartile = 57) and 38.6 (median: 37, lower quartile: 20, upper quartile: 56) for the training and testing datasets respectively.

For a 10-year increase in age, risk for any cancer increased by 72% (95% confidence interval [CI] of Hazard Ratio [HR]: [1.71, 1.72]) and 74% (95% CI HR: [1.73, 1.76]) in the training and testing datasets, respectively. The datasets had an even distribution of males and females. Males had a 39% (95% CI: [1.34, 1.41]) and 40% (95% CI: [1.39, 1.42]) increased risk for any cancer versus females in the training and testing datasets respectively. The mean follow-up time in both datasets was 1,983 days (median = 2,009 days) in both the training and testing datasets.

For a 10-year increase in age, risk for colon cancer increased by 93% (95% CI: [1.91, 1.96]) and 97% (95% CI: [1.95, 1.99]) in the training and testing datasets respectively. The datasets had an even distribution of males and females. Males had a 28% (95% CI: [1.21,1.34]) and 24% (95% CI: [1.20, 1.30]) increased risk for colon cancer versus females in the training and testing datasets respectively. The mean follow-up time was 2,000 days (median = 2,009) and 2,002 (median = 2,009) in the training and testing datasets respectively.

For a 10-year increase in age, risk for prostate cancer increased by 83% (95% CI of HR: [1.82, 1.84] and 102% (95% CI of HR: [2.01, 2.04]) in the training and testing datasets, respectively. The mean follow-up time was 1,998 days (median = 2,009) and 1,999 (median = 2,009) in the training and testing datasets respectively.

For a 10-year increase in age, risk for breast cancer increased by 46% (95% CI of HR: [1.45, 1.47] and 50% (95% CI of HR: [1.48, 1.51]) in the training and testing sets respectively. The mean follow-up time was 2,001 days (median = 2,009) and 2,002 days (median = 2009) in the training and testing datasets respectively.

### Systematic scan of medications associated with any cancer risk: Cox model

**Figure 2:**
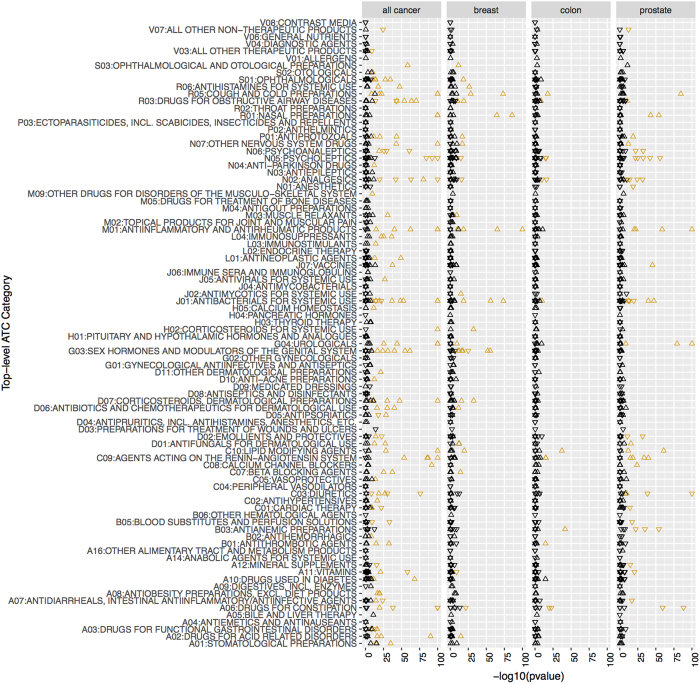
Manhattan plots (−log10(p-value)) for each drug categorized by Anatomical Therapeutic Chemical anatomical main group) in Cox analyses by cancer type. Orange color denotes tentative signals. P-values lower than 1 × 10^−100^ set to 1 × 10^−100^ for clarity. Upward triangles indicate HR >1 and downward triangles HR <1.

We discuss results for each cancer type, starting with any cancer. In our Cox analysis for longitudinal any cancer risk, 158 drugs were statistically significantly associated with cancer risk after adjustment for multiple hypotheses (Bonferroni threshold p < 9 × 10^−5^) in the training set (29% of all 552 types of drugs) and 177 were statistically significantly associated with cancer risk (p < 9 × 10^−5^) in the testing set (32% of all 552). Of these, 141 (26%) were statistically significant (p < 9 × 10^−5^ and HR consistent) in both datasets and are therefore considered as tentative signals (Figure S1 and Fig. 2). The number of findings achieving statistical significance was much greater than what we would expect if no drugs were correlated with time to cancer (Figure S2). The effect sizes in training and testing datasets were correlated (ρ = 0.33, p = 5 × 10^−15^, Figure S3). As expected, the −log10(p-values) were also highly correlated (ρ = 0.95). Most (368 of 552) of the HRs were greater than 1 (median 1.17, IQR 0.90–0.1.41) and most of the HRs representing tentative signals (109 of 141) were also greater than 1 (median 1.26, IQR 1.13-1.4), i.e. it was more frequent to find putative adverse associations rather than associations with decreased cancer risk (Figures S3 and S4). 55 out of 88 ATC level 1 categories (representing anatomical main group) had at least one tentative signal (Fig. 2).

### Systematic scan of medications associated with breast cancer risk: Cox model

We now discuss breast cancer. In our Cox analysis for longitudinal breast cancer risk, 56 drugs were statistically significantly associated with breast cancer risk after adjustment for multiple hypotheses (Bonferroni threshold p < 9 × 10^−5^) in the training set (10% of all 552 types of drugs) and 56 were statistically significantly associated with cancer risk (p < 9 × 10^−5^) in the testing set (10% of all 552). Of these, 41 (7%) were statistically significant (p < 9 × 10^−5^ and HR consistent) in both datasets and are therefore considered as tentative signals (Figure S1 and Fig. 2). The number of findings achieving statistical significance was much greater than what we would expect if no drugs were correlated with time to cancer (Figure S2). The effect sizes in training and testing datasets were correlated (ρ = 0.39, p = 1 × 10^−15^, Figure S3). The −log10(p-values) were also modestly correlated (ρ = 0.57, 1 × 10^−46^). Most (302 of 552) of the HRs were greater than 1 (median 1.09, IQR 0.53-1.46) and most of the HRs representing tentative signals (36 of 41) were also greater than 1 (median 1.46, IQR 1.3-1.6), i.e. it was more frequent to find putative adverse associations rather than associations with decreased cancer risk (Figures S3 and S4). 25 out of 88 ATC level 1 categories (representing anatomical main group) had at least one tentative signal (Fig. 2).

### Systematic scan of medications associated with prostate cancer risk: Cox model

We now discuss prostate cancer. In our Cox analysis for longitudinal prostate cancer risk, 67 drugs were statistically significantly associated with prostate cancer risk after adjustment for multiple hypotheses (Bonferroni threshold p < 9 × 10^−5^) in the training set (12% of all 552 types of drugs) and 74 were statistically significantly associated with cancer risk (p < 9 × 10^−5^) in the testing set (13% of all 552). Of these, 56 (10%) were statistically significant (p < 9 × 10^−5^ and HR consistent) in both datasets and are therefore considered as tentative signals (Figure S1 and Fig. 2). The number of findings achieving statistical significance was much greater than what we would expect if no drugs were correlated with time to cancer (Figure S2). The effect sizes in training and testing datasets were correlated (ρ = 0.49, p = 1 × 10^−32^, Figure S3). The −log10(p-values) were also modestly correlated (ρ = 0.65, 1 × 10^−66^). 60% (329 of 552) of the HRs were less than 1 (median 0.9, IQR 0.3-1.3) and most of the HRs representing tentative signals (30 of 56) were also less than 1 (median 0.8, IQR 0.5-1.4), i.e. it was more frequent to find putative protective associations rather than associations with decreased cancer risk (Figures S3 and S4). 27 out of 88 ATC level 1 categories (representing anatomical main group) had at least one tentative signal (Fig. 2).

### Systematic scan of medications associated with colon cancer risk: Cox model

Of all the cancers tested, colon cancer had the least number of tentative signals. 19 drugs were statistically significantly associated with colon cancer risk after adjustment for multiple hypotheses (Bonferroni threshold p < 9 × 10^−5^) in the training set (3% of all 552 types of drugs) and 27 were statistically significantly associated with cancer risk (p < 9 × 10^−5^) in the testing set (5% of all 552). Of these, 14 (3%) were statistically significant (p < 9 × 10^−5^ and HR consistent) in both datasets and are therefore considered as tentative signals (Figure S1 and Fig. 2). The number of findings achieving statistical significance was much greater than what we would expect if no drugs were correlated with time to cancer (Figure S2). The effect sizes in training and testing datasets were correlated (ρ = 0.50, p = 2 × 10^−35^, Figure S3). The −log10(p-values) were also modestly correlated (ρ = 0.56, 5 × 10^−47^). 57% (314 of 552) of the HRs were less than 1 (median 0.9, IQR 0.5-1.2). Conversely, 8 of 14 of the tentative signals were greater than 1 (median 1.3, IQR 0.5-1.5). (Figures S3 and S4). 10 out of 88 ATC level 1 categories (representing anatomical main group) had at least one tentative signal (Fig. 2).

### Systematic scan of medications in any cancer cases: case-crossover findings

**Figure 3:**
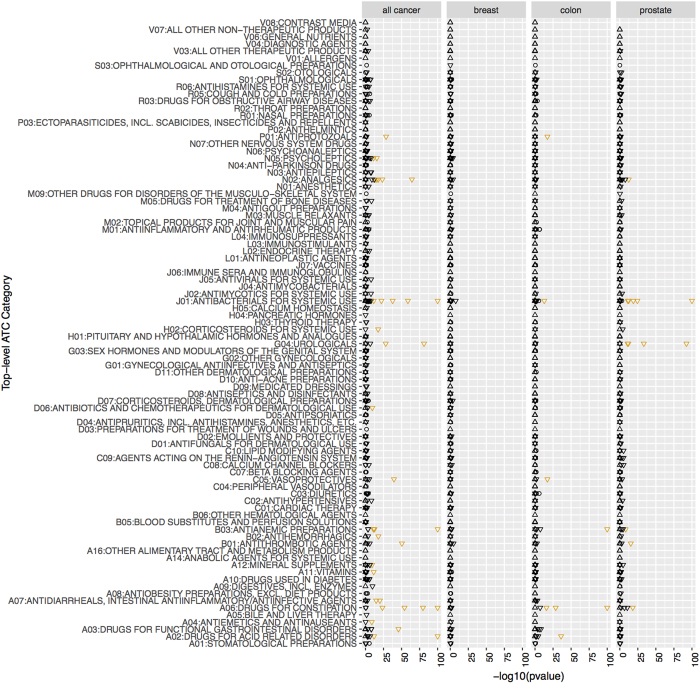
Manhattan plot (−log10(p-value)) for each drug categorized by Anatomical Therapeutic Chemical anatomical main group) in case-crossover analyses. Orange color denotes tentative signals. P-values lower than 1 × 10^−100^ set to 1 × 10^−100^ for clarity. Upward triangles indicate OR >1 and downward triangles OR <1.

In the case-crossover analyses, 48 drugs were statistically significantly associated with any cancer risk after adjustment for multiple hypotheses (Bonferroni threshold p < 9 × 10^−5^) in the training set (9% of all 552 drugs) and 50 were statistically significantly associated with cancer risk (p < 9 × 10^−5^) in the testing set (9% of all 552) (Figure S5 and Fig. 3). The number of statistically significant findings were much greater than what we would expect if no drugs were associated with cancer risk (Figure S6). Of these, 36 (7%) were tentative signals, or statistically significant (p < 9 × 10^−5^ and OR consistent) in both training and testing datasets. The effect sizes in training and testing datasets were correlated (ρ = 0.41 [p = 6 × 10^−15^]). As expected, the −log10(p-values) were also highly correlated (ρ = 0.93). The odds ratios (OR) were centered at 0.88 (median 0.88, IQR 0.72-1.04). All of the ORs representing tentative signals were less than 1 (median 0.60, IQR 0.49-0.72), i.e. they represented protective associations (Figure S7). 18 out of 88 ATC level 1 categories (representing anatomical main group) had at least one tentative signal (Fig. 3).

### Systematic scan of medications in breast cancer cases: case-crossover findings

No drugs were statistically significantly associated with breast cancer risk after adjustment for multiple hypotheses (Bonferroni threshold p < 9 × 10^−5^) in the training set and 2 were statistically significantly associated with cancer risk (p < 9 × 10^−5^) in the testing set (0.4% of all 552) (Figure S5 and Fig. 3). The effect sizes in training and testing datasets were not correlated (ρ = 0.003 [p = 0.96]).

### Systematic scan of medications in breast cancer cases: case-crossover findings

16 drugs were statistically significantly associated with any cancer risk after adjustment for multiple hypotheses (Bonferroni threshold p < 9 × 10^−5^) in the training set (3% of all 552 drugs) and 15 were statistically significantly associated with cancer risk (p < 9 × 10^−5^) in the testing set (3% of all 552) (Figure S5 and Fig. 3). Of these, 13 (2%) were tentative signals, or statistically significant (p < 9 × 10^−5^ and OR consistent) in both training and testing datasets. The effect sizes in training and testing datasets were correlated (ρ = 0.46 [p = 7 × 10^−13^]). As expected, the −log10(p-values) were also correlated (ρ = 0.27). The odds ratios (OR) were centered at 0.87 (median 0.87, IQR 0.67-1.02). Like in any cancer, all of the ORs representing tentative signals were less than 1 (median 0.47, IQR 0.32-0.61), i.e. they represented protective associations (Figure S7). 6 out of 88 ATC level 1 categories (representing anatomical main group) had at least one tentative signal (Fig. 3).

### Systematic scan of medications in colon cancer cases: case-crossover findings

13 drugs were statistically significantly associated with colon cancer risk after adjustment for multiple hypotheses (Bonferroni threshold p < 9 × 10^−5^) in the training set (2% of all 552 drugs) and 11 were statistically significantly associated with cancer risk (p < 9 × 10^−5^) in the testing set (2% of all 552) (Figure S5 and Fig. 3). The number of statistically significant findings were much greater than what we would expect if no drugs were associated with cancer risk (Figure S6). Of these, 8 (1%) were tentative signals, or statistically significant (p < 9 × 10^−5^ and OR consistent) in both training and testing datasets. However, the effect sizes in training and testing datasets were not correlated (ρ = 0.03 [p = 0.74]). The −log10(p-values) were modestly correlated (ρ = 0.17, p = 0.03). The odds ratios (OR) were centered at 0.93 (median 0.93, IQR 0.70-1.16). Like in any and colon cancers, all of the ORs representing tentative signals were less than 1 (median 0.35, IQR 0.18-0.41), i.e. they represented protective associations (Figure S7). 6 out of 88 ATC level 1 categories (representing anatomical main group) had at least one tentative signal (Fig. 3).

### Concordance between the two analytical approaches in each cancer type

We discuss concordance between the Cox and case-crossover analyses for each of the site-specific and any cancer separately. First, we begin with any cancer. Of the 141 and 36 tentative signals found in the Cox and case-crossover analyses respectively in any cancer, 26 were tentative signals in both analyses (were tentatively validated in both training and testing datasets). Of those 26, only 12 (46%) had effect sizes that were concordant (both below 1, suggesting decreased cancer risk) (Table 1); another 14 had effects in opposite direction (increased risk in Cox models, decreased risk in case-crossover models) (Table 2). Of the 115 tentative signals seen only in the Cox analysis, 63 had an effect estimate in the same direction in the case-crossover analysis (n = 46 increased risk, n = 17 decreased risk), although the results of the case-crossover analyses did not meet the statistical/validation thresholds required. Of the 10 tentative signals seen in the case-crossover analysis, 7 also had an effect estimate in the same direction in the Cox model (all 7 decreased risk), although the results of the Cox analyses did not meet the statistical/validation thresholds required. Overall, tentative signals emerging from either the Cox or the case-crossover analyses had effects in the same direction more frequently than in opposite direction (82 vs. 67, kappa of 0.24, p = 1 × 10^−5^).

For prostate cancer, of the 56 and 13 tentative signals found in the Cox and case-crossover analyses respectively in prostate cancer, 8 were tentative signals in both analyses (were tentatively validated in both training and testing datasets). Of those 8, 4 (50%) had effect sizes that were concordant (both below 1, suggesting decreased cancer risk) (Table 3); another 4 had effects in opposite direction (increased risk in Cox models, decreased risk in case-crossover models) (Table 4). Of the 48 tentative signals seen only in the Cox analysis, 30 had an effect estimate in the same direction in the case-crossover analysis (n = 9 increased risk, n = 21 decreased risk), although the results of the case-crossover analyses did not meet the statistical/validation thresholds required. Of the 5 tentative signals seen only in the case-crossover analysis, 3 also had an effect estimate in the same direction in the Cox model (all 3 decreased risk), although the results of the Cox analyses did not meet the statistical/validation thresholds required. Overall, tentative signals emerging from either the Cox or the case-crossover analyses had associations in the same direction more frequently than in opposite direction (37 vs. 22, kappa of 0.23, p = 0.03).

For colon cancer, of the 14 and 13 tentative signals found in the Cox and case-crossover analyses respectively in colon cancer, 2 were tentative signals in both analyses (were tentatively validated in both training and testing datasets). Of those 2, 1 (50%) had effect sizes that were concordant (below 1, suggesting decreased cancer risk) (Table 5, first row); the other one had association in the opposite direction (increased risk in Cox models, decreased risk in case-crossover models) (Table 5, second row). Of the 14 tentative signals seen only in the Cox analysis, 6 had an effect estimate in the same direction in the case-crossover analysis (n = 4 increased risk, n = 2 decreased risk), although the results of the case-crossover analyses did not meet the statistical/validation thresholds required. Of the 5 tentative signals seen only in the case-crossover analysis, 2 also had an effect estimate in the same direction in the Cox model (all 2 decreased risk), although the results of the Cox analyses did not meet the statistical/validation thresholds required. Overall, we could not conclude that tentative signals emerging from either the Cox or the case-crossover analyses had associations in the same direction more frequently than in opposite direction (9 vs. 9, kappa of 0.13, p = 0.5).

For breast cancer, we found 41 tentative signals in the Cox models but 0 tentative signals in the case-crossover models. Of the 41 tentative signals seen in the Cox analysis, 24 had an association in the same direction as the case-crossover analysis (n = 22 increased risk, n = 2 decreased risk) and the kappa was non-significant (p = 0.96).

### Comparison of medication-wide findings with previously reported findings of cancer risk

We checked the associations between drugs previously hypothesized to be associated with any, breast, colon, or prostate cancer risk as reported in ref. 13, (Table 6). These drugs included thiazolidones, insulin, angiotensin converting enzyme inhibitors (ACEis), diuretics, tumor necrosis factor inhibitors (TNFis), angiotensin II receptor blockers (ARBs), and prednisolone-type corticosteroids. Because there are many diuretics that can be chosen, we observed the report all the findings for the category of drugs belonging to diuretics (n = 7), totaling 14 drugs considered.

For any cancer, we found 8 of the 14 drugs had a tentative signal in the Cox analyses, and 6/8 had an increased cancer risk. In the case-crossover analysis, 1 drug had a tentative signal for decreased risk. The direction of effect estimate was concordant in the two analyses for 7 of the 14 but tentative signals passing required significance thresholds were seen only for one drug that actually had a decreased risk for cancer (Table 6). Thiazolidinediones, diuretics, ACE inhibitors, and potassium-sparing agents had increased risk estimates in both analyses, but they passed the required significance threshold only in the Cox analyses.

There was less concordance between the site-specific cancers (ie, prostate, colon, and breast) and previously reported findings. For example, angiotensin II receptor blockers (ARBs) had a tentative signal for the Cox analyses in both prostate and colon cancers (Table 6). Furthermore, several diuretics also had a tentative signal in the Cox analysis in prostate cancer. We did not observe tentative signals for both Cox and case-crossover analyses for any of the site-specific cancers for previously reported drugs.

### Differences in category- and drug-level associations in any cancer

Figure S8 depicts differences between category- and drug-level associations in any cancer. Most category-level associations had the same sign of association as the majority of the drugs in that category. However, within specific categories, we had little evidence to support specificity of associations by drug action. For example, in category C07 (“Beta-blocking agents”), we observed no qualitative difference in direction of HRs in any cancer for tentative signals for drugs that putatively function differently, such as selective vs. non-selective beta-blocking agents (C07AA and C07AB respectively, Figure S7, in red box). Another example is in category C10 (“lipid modifying agents”) is lack of difference between tentative signals for statins (C10AA) and fibrates (C10AX).

## Discussion

Here, we have implemented a systematic investigation of all available prescription drugs in association with cancer risk in a national population-wide evaluation. We set out to find drugs tentatively linked with any cancer (as well as site-specific cancers) in an observational setting; however, we have found that in our analyses of a large country-wide cohort, 26% of all drugs are associated with decreased or increased any cancer risk in a longitudinal Cox analysis framework. Site-specific cancers could not explain the majority of associations and we found 10, 7, and 3% of drugs queried were associated with prostate, breast, and colon cancer respectively. When utilizing methods to account for time-invariant confounding, the case-crossover approach, 7% of drugs were associated with only decreased any cancer risk. When combining both approaches, 2% remain associated with decreased any cancer risk. Of 9 drugs proposed by at least one previous meta-analysis in the literature to be associated with cancer risk, thiazolidinediones and three classes of antihypertensives had increased risk estimates in both Cox and case-crossover analyses, but they passed the required significance threshold only in the former. Very few drugs were found in the case-crossover analyses for the site-specific cancers and therefore we found little overlap between the two analytic approaches.

Nevertheless, detected pharmacoepidemiological associations of cancer risk are likely to be a mixture of some true-positive cancer associations and false-positive cancer associations^2^. False-positives in our prescription-wide approach are likely to reflect biases such as survivorship bias and confounding (e.g., confounding by indication) as our analytical approach excludes other biases such as selective analysis and outcome reporting, which may be also major problems in pharmacoepidemiologic studies that report on one or a few drugs at a time. Estimates of associations (effect sizes and p-values) require more than just multiplicity adjustments for their interpretation. Significance testing assumes a theoretical null distribution (no association between drugs and outcomes) and we observed a large deviation from the theoretical null (Figure S3). Schuemie and colleagues argue for calibrating p-values empirically against what is actually observed on “negative control” drugs^21^,^22^. For example, if there are large effects or small p-values for drugs not known to be associated with the outcome of interest, this information may be used to re-adjust the p-values based on the theoretical null. Relatedly, Prasad and Jena propose “pre-specified falsification endpoints”, whereby researchers test pre-specified drugs that are highly unlikely to be linked to the outcome of interest^23^. If these falsification hypotheses are associated with the outcome then researchers can conclude that characteristics underlying the population may be biasing also other associations that may be probed in the same dataset. However, both exercises (e.g., calibrating p-values and determining pre-specified falsification hypotheses) require a database of drugs that are known to be definitively not linked to the outcome, in this case cancer. Furthermore, omitted variable bias, or unaccounted confounding, can result in highly significant but spurious results in observational studies^24^. Even though most drugs probably do not increase or decrease cancer risk, we do not have large-scale mega-trials with long-term follow-up and meticulous cancer ascertainment that can offer solid evidence of no association of specific drugs with cancer, and small effects on cancer risk cannot be excluded with high certainty.

We attempted to correlate our large-scale “agnostic” findings with claims about increased risk in published meta-analyses in the literature and we found some concordant signals with Cox analyses, but not so with case-crossover analyses. It is possible that drugs that were previously proposed to be associated with increased cancer risk in single meta-analyses represent largely random associations and may be false-positives due to confounding by indication or other biases. Alternatively, some of the findings in our systematic scan may be false-negatives, especially in the case-crossover analysis. This would mean that effect sizes are small or modest at best. The previous umbrella review (ref. 13) showed that whenever a significant association with increased cancer risk had been published in one meta-analysis, there almost always existed other meta-analyses that showed no increased or even decreased cancer risk for the same drug. Thus, this picture is overall consistent with a large prevalence of false-positive claims in cancer pharmacoepidemiology, although a few genuine signals are possible.

We found modest concordance between the Cox analysis and the case-crossover approach in any cancer; however, we found little concordance between the approaches in site-specific cancers. While the Cox and case-crossover models are symmetric in several aspects/assumptions, they are not identical in all their assumptions. We acknowledge that these models are testing different carcinogenesis mechanisms. Both models exclude the first year after the first half-year after surveillance; however, the Cox model considers that the cancer risk is activated after that window and remains activated after that point until the end of surveillance. Conversely, the case-crossover model assumes that it is likely to have a 1- to 2-year time lag from exposure to cancer rather than 0 to 1 year from exposure to cancer. Few medications fit both paths/mechanisms and none fit both paths with increased cancer risk, when considering the statistical/validation thresholds. However, there are many drugs with estimates of effect in the same direction with both models.

There are major limitations to using the case-crossover approach in investigation of drug associations in cancer. First, the method is best suited if the effect is immediate and/or transient and the outcome is acute^19^. In our investigation, we used the method to detect non-acute and non-transient associations between drugs and cancer. Furthermore, the case-crossover approach is sensitive to exposure time windows and window length can bias estimates^25^. Second, selection bias can occur if the drug prescription rate in the reference period (one year window immediately prior to cancer diagnosis) is not representative of the hazard period (one year window and one year prior to cancer). We emphasize the reason we implement the case-crossover approach is to demonstrate how findings may change due to modeling choices.

The tentative signals with increased cancer risk were far fewer than the consistent signals with decreased cancer risk in the case-crossover analysis. The latter group may be spurious and may be due to survivorship bias. Moreover, associations with decreased cancer risk are likely to be clinically non-useful. It is unlikely that healthy people would take a medication that is aimed to be used for specific diseases and may have cost and other toxicities with the intent of decreasing cancer risk in the long term. Conversely, increased risk associations are more clinically informative and, if genuine, they may affect the decision-making about the use of specific drugs.

Other biases include confounding by indication, protopathic bias, and ascertainment biases are well documented for pharmacoepidemiological investigation by the ENCEPP^14^. For example, confounding by indication is characterized by drugs being prescribed to individuals who are at risk for cancer. For example, smoking is a risk factor for many diseases, such as chronic obstructive pulmonary disorder (COPD) and lung cancer. A drug prescribed for COPD may falsely be associated with cancer due to the shared risk factor. Relatedly, drugs may also be prescribed for indications that are symptoms of future cancer. This type of confounding bias is known as protopathic bias that may explain the association between PPIs and cancer described above. Relatedly, ascertainment biases lead to individuals who are more prone to other illnesses to be diagnosed with cancer or prescribed at a higher rate with drugs due to more frequent and/or longer duration of clinical care. However it remains elusive in any epidemiological study how to discriminate biased findings from true signals^26^. For example, it is uncertain whether one would be able or willing to discard a “positive” finding indicating an adverse result that they have found by attributing it to such biases, let alone that they will not run an analysis because they have pre-emptively considered these biases and have decided to not perform the study.

The primary strengths of our study includes its systematic evaluation of 552 types of prescribed drugs in relation to longitudinal cancer risk (over 5 years of follow-up) in a large cohort with optimal power, the entire population of Sweden. Nevertheless, our study does have some drawbacks. First, we considered each drug separately for its putative association with cancer, assuming that drug prescriptions were not correlated with one another, while it is plausible that multiple drugs are being prescribed for the same indication. By potentially assessing multiple drugs concurrently, one can ascertain the independent risk for each after consideration of other co-prescribed ones. Nevertheless, the testing of each drug type separately approximates better the way that pharmacoepidemiological studies have functioned to-date, trying to dissect associations with one or a few drugs only at a time. Relatedly, we acknowledge using a conservative approach for correcting multiple hypotheses or the family-wide error rate, the Bonferroni correction. While our analysis in any cancer is indeed well-powered to detect associations for our sample sizes, other methods for considering multiple hypotheses are indeed available, such as the False Discovery Rate (FDR)^17^. Signals may be further refined by filtering by association size. Second, our approach did not incorporate any biological plausibility considerations, which may be considered important in specific settings. We suggest that for the strongest tentative signals, careful consideration of biological mechanisms and additional tailored analyses may be useful to probe them further. However, it is best for these to be pre-specified by interested pharmacoepidemiologists, since otherwise plausibility and adjusted analyses can lead to spurious results due to selective analyses reporting. Third, while the ATC classification intends to divide drugs into their pharmacological, chemical, and therapeutic subgroups, the most specific ATC assignment can be heterogeneous. For example, the code G04FA describes a group of drugs used for erectile dysfunction but are different in their chemical composition and/or mode of action (e.g., alprostadil, papverine, sildenafil are different drugs that are all assigned to the ATC code G04FA). Fourth, the drug dose and duration of use are not available and therefore not taken into account in our analyses. We acknowledge that we do not have the opportunity to test cancer risk as a function of dose. Fifth, we acknowledge that the follow-up time in our study is relatively short. Our surveillance time to query for drugs in future cancer risk is only 5 years and we acknowledge that exposure to putative causative agents may be those that (repeatedly) occur much before the surveillance window. Relatedly, and sixth, the design of our longitudinal study may influence results. In our Cox analyses, to ensure removal of prevalent cancer cases and drug exposures, we imposed a 180 window from the start of surveillance to begin querying for drugs associated with cancer. Further, it is hypothesized that a causal link between exposure and common cancers may have a longer latency period, so we also imposed that drug exposure must come at least one year before the event in both case-crossover and Cox analyses (and potential exposures that occur just one year prior to diagnoses are not counted) to capture chronic and non-acute associations in cancer. However, by not capturing all possible exposures even up to the cancer diagnosis may lead to differential misclassification leading to an over or under-estimated association size.

Seventh, our comparisons of our findings with an umbrella meta-analysis of clinical trial surveillance investigations are not wholly fair: some of the findings report site-specific risk (e.g., bladder cancer) and consider drugs in coded in a different form (e.g., not the ATC code utilized in this study). Despite differences in study scenarios, it is a way of ascertaining the differences and similarities between the current-day documented pharmacoepidemiological findings. Eighth, while we make an attempt to document signals that are tentatively replicated (found in two cohorts living in two different geographic regions in Sweden), ideally findings replicated in another country and dataset would be less likely to be spurious or an artifact of country-specific phenomena. We propose future studies to interrogate findings that are concordant (and discordant) in other cancer registries linked with drug information.

This investigation provides another way in the growing arsenal of methods to search for drugs associated with an adverse outcome such as cancer risk. A key concern in pharmacoepidemiology and drug surveillance remains how often do we attain, and the balance between, false-positive and false-negative results^27^. A major advantage of evaluating large-scale national population-wide data is optimal power. However, in the end, we show the ease by which associations can be found in large-scale population databases resulting in an implausibly large number of the prescribed drugs associated with the outcome, indicative of false-positive findings due to bias such as confounding. This may be a more prominent problem with associations of decreased, rather than increased, cancer risk, but this hypothesis requires testing in other large population registries. Because observational studies are at present the primary means of evaluating the possible effects of drugs after they enter the market, we will need to continue to develop methods and studies extending the method described here to identify and mitigate these drawbacks^27^. One way would be to use new methods to adjust for all prescriptions simultaneously in models that can accommodate both large sample sizes and drug variables (e.g. ref. 28). In the meanwhile, interpretation of significant associations of cancer risk from observational studies needs to be cautious.

## Additional Information

**How to cite this article**: Patel, C. J. *et al*. Systematic assessment of pharmaceutical prescriptions in association with cancer risk: a method to conduct a population-wide medication-wide longitudinal study. *Sci. Rep*. **6**, 31308; doi: 10.1038/srep31308 (2016).

## Supplementary Material

Supplementary Information

## Figures and Tables

**Table 1:** Tentative signals in Cox and Case-crossover analyses in any cancer with consistent direction of effect.

Drug Description	Category Description	Cox HR	Cox P	CC OR	CC P
Propulsives	DRUGS FOR FUNCTIONAL GASTROINTESTINAL DISORDERS	0.81 [0.77, 0.86]	3 × 10^−14^	0.44 [0.39, 0.49]	2 × 10^−46^
Contact laxatives	DRUGS FOR CONSTIPATION	0.54 [0.52, 0.57]	1 × 10^−100^	0.46 [0.42, 0.51]	9 × 10^−55^
Osmotically acting laxatives	DRUGS FOR CONSTIPATION	0.79 [0.77, 0.80]	1 × 10^−100^	0.45 [0.43, 0.47]	1 × 10^−100^
Enemas	DRUGS FOR CONSTIPATION	0.70 [0.67, 0.74]	1 × 10^−38^	0.32 [0.29, 0.36]	2 × 10^−80^
Antibiotics	ANTIDIARRHEALS, INTESTINAL ANTIINFLAMMATORY AGENTS	0.77 [0.74, 0.81]	1 × 10^−24^	0.64 [0.57, 0.71]	2 × 10^−15^
Vitamin B-complex, other combinations	VITAMINS	0.54 [0.50, 0.58]	8 × 10^−59^	0.55 [0.46, 0.65]	4 × 10^−12^
Potassium	MINERAL SUPPLEMENTS	0.84 [0.80, 0.88]	3 × 10^−16^	0.73 [0.66, 0.81]	7 × 10^−10^
Trimethoprim and derivatives	ANTIBACTERIALS FOR SYSTEMIC USE	0.90 [0.88, 0.93]	5 × 10^−15^	0.81 [0.76, 0.86]	4 × 10^−12^
Phenylpiperidine derivatives	ANALGESICS	0.43 [0.40, 0.46]	1 × 10^−100^	0.54 [0.46, 0.64]	4 × 10^−14^
Diphenylpropylamine derivatives	ANALGESICS	0.88 [0.86, 0.90]	2 × 10^−20^	0.72 [0.68, 0.77]	4 × 10^−24^
Oripavine derivatives	ANALGESICS	0.54 [0.49, 0.59]	9 × 10^−43^	0.49 [0.42, 0.58]	2 × 10^−16^
Benzodiazepine derivatives	PSYCHOLEPTICS	0.79 [0.77, 0.81]	4 × 10^−85^	0.79 [0.74, 0.83]	2 × 10^−16^

Drug description is in second column. HR = Hazard Ratio, p = pvalue of association, OR = Odds Ratio, CC = Case Crossover.

**Table 2:** Tentative signals in Cox and case-crossover analyses that had opposite direction of effect sizes in any cancer.

Drug Description	Category Description	Cox HR	Cox P	CC OR	CC P
Proton pump inhibitors	DRUGS FOR ACID RELATED DISORDERS	1.19 [1.17, 1.21]	8 × 10^−91^	0.64 [0.62, 0.67]	1 × 10^−100^
Combinations for eradication of Helicobacter pylori	DRUGS FOR ACID RELATED DISORDERS	1.37 [1.28, 1.46]	2 × 10^−22^	0.60 [0.52, 0.69]	1 × 10^−12^
Bulk-forming laxatives	DRUGS FOR CONSTIPATION	1.16 [1.12, 1.20]	3 × 10^−20^	0.68 [0.64, 0.74]	3 × 10^−24^
Heparin group	ANTITHROMBOTIC AGENTS	1.13 [1.10, 1.16]	2 × 10^−15^	0.58 [0.54, 0.63]	3 × 10^−51^
Amino acids	ANTIHEMORRHAGICS	1.21 [1.13, 1.29]	8 × 10^−9^	0.51 [0.44, 0.59]	1 × 10^−18^
Corticosteroids	VASOPROTECTIVES	1.14 [1.10, 1.18]	2 × 10^−14^	0.60 [0.55, 0.65]	4 × 10^−40^
Antivirals	ANTIBIOTICS AND CHEMOTHERAPEUTICS FOR DERMATOLOGICAL USE	1.54 [1.43, 1.66]	8 × 10^−31^	0.62 [0.53, 0.72]	3 × 10^−10^
Alpha-adrenoreceptor antagonists	UROLOGICALS	1.62 [1.56, 1.68]	1 × 10^−100^	0.48 [0.44, 0.51]	8 × 10^−82^
Testosterone-5-alpha reductase inhibitors	UROLOGICALS	1.36 [1.30, 1.42]	2 × 10^−44^	0.58 [0.52, 0.64]	8 × 10^−29^
Glucocorticoids	CORTICOSTEROIDS FOR SYSTEMIC USE	1.28 [1.25, 1.30]	1 × 10^−100^	0.80 [0.77, 0.84]	3 × 10^−18^
Penicillins with extended spectrum	ANTIBACTERIALS FOR SYSTEMIC USE	1.07 [1.05, 1.09]	5 × 10^−15^	0.82 [0.79, 0.86]	8 × 10^−23^
Fluoroquinolones	ANTIBACTERIALS FOR SYSTEMIC USE	1.16 [1.14, 1.19]	3 × 10^−53^	0.49 [0.47, 0.51]	1 × 10^−100^
Other opioids	ANALGESICS	1.20 [1.18, 1.22]	5 × 10^−81^	0.81 [0.77, 0.85]	2 × 10^−18^
Nitroimidazole derivatives	ANTIPROTOZOALS	1.26 [1.22, 1.30]	8 × 10^−44^	0.64 [0.59, 0.69]	3 × 10^−29^

Drug description is in second column. HR = Hazard Ratio, p = pvalue of association, OR = Odds Ratio, CC = Case-crossover.

**Table 3:** Tentative signals in Cox and case-crossover analyses that had consistent direction of effect sizes in prostate cancer.

Drug Description	Category Description	Cox HR	Cox P	CC OR	CC P
Osmotically acting laxatives	DRUGS FOR CONSTIPATION	0.61 [0.58, 0.64]	4.E-89	0.61 [0.55, 0.68]	1.E-18
Iron bivalent, oral preparations	ANTIANEMIC PREPARATIONS	0.48 [0.44, 0.53]	7.E-55	0.53 [0.43, 0.66]	7.E-09
Trimethoprim and derivatives	ANTIBACTERIALS FOR SYSTEMIC USE	0.66 [0.60, 0.72]	3.E-20	0.43 [0.36, 0.52]	2.E-19
Nitrofuran derivatives	ANTIBACTERIALS FOR SYSTEMIC USE	0.50 [0.42, 0.60]	7.E-14	0.25 [0.17, 0.36]	6.E-13

Drug description is in second column. HR = Hazard Ratio, p = pvalue of association, OR = Odds Ratio, CC = Case-crossover.

**Table 4:** Tentative signals in Cox and case-crossover analyses that had opposite direction of effect sizes in prostate cancer.

Drug Description	Category Description	Cox HR	Cox P	CC OR	CC P
Drugs used in erectile dysfunction	UROLOGICALS	2.08 [1.98, 2.18]	1.00E-100	0.65 [0.57, 0.73]	1.48E-12
Alpha-adrenoreceptor antagonists	UROLOGICALS	1.70 [1.61, 1.79]	3.94E-80	0.32 [0.29, 0.36]	4.27E-93
Testosterone-5-alpha reductase inhibitors	UROLOGICALS	1.22 [1.14, 1.31]	4.99E-09	0.40 [0.34, 0.46]	4.74E-34
Fluoroquinolones	ANTIBACTERIALS FOR SYSTEMIC USE	1.16 [1.11, 1.20]	5.88E-13	0.29 [0.26, 0.31]	1.00E-100

Drug description is in second column. HR = Hazard Ratio, p = pvalue of association, OR = Odds Ratio, CC = Case-crossover.

**Table 5:** Tentative signals in Cox and case-crossover analyses that had a consistent (row 1) and opposite direction (row 2) of effect sizes in colon cancer.

Drug Description	Category Description	Cox HR	Cox P	CC OR	CC P
Osmotically acting laxatives	DRUGS FOR CONSTIPATION	0.70 [0.65, 0.75]	3.E-23	0.14 [0.12, 0.16]	1.E-100
Iron bivalent, oral preparations	ANTIANEMIC PREPARATIONS	1.72 [1.60, 1.87]	2.E-42	0.20 [0.18, 0.23]	1.E-100

Drug description is in second column. HR = Hazard Ratio, p = pvalue of association, OR = Odds Ratio, CC = Case-crossover.

**Table 6:** Lookup of prescription-wide findings of candidate cancer associated drugs in meta-analyses.

Meta-analysis Drug Description	Prescription-wide Description (ATC Code)	Meta-analysis RR/RR (Cancer Type)	Any Cancer: in Cox and/or CC	Prostate Cancer: Signal in Cox and/or CC	Colon Cancer: Signal in Cox and/or CC	Breast Cancer: Signal in Cox and/or CC
Insulin	Insulins and analogues for injection, fast-acting (A10AB)	1.38, 1.5 (Colorectal), 2.2, 4.8 (Pancreatic)	Cox/CC	None	None	None
Pioglitazone/Thiaglitazone	Thiazolidinediones (A10BG)	1.15 (Bladder Cancer)	Cox	None	None	None
ARB	Angiotensin II antagonists, plain (C09CA)	1.1, 1 (all)	Cox	Cox	Cox	None
ACE Inhibitors	ACE inhibitors, plain (C09AA)	1.1, 1 (all)	Cox	Cox	None	None
Diuretics	Thiazides, plain (C03AA)	0.9 (all), 1.4 (renal cancer)	Cox	None	None	None
Diuretics	Thiazides and potassium in combination (C03AB)	0.9 (all), 1.4 (renal cancer)	None	None	None	None
Diuretics	Sulfonamides, plain (C03BA)	0.9 (all), 1.4 (renal cancer)	Cox	None	None	None
Diuretics	Sulfonamides, plain (C03CA)	0.9 (all), 1.4 (renal cancer)	Cox	Cox	None	None
Diuretics	Aldosterone antagonists (C03DA)	0.9 (all), 1.4 (renal cancer)	Cox	Cox	None	None
Diuretics	Other potassium-sparing agents (C03DB)	0.9 (all), 1.4 (renal cancer)	None	None	None	None
Diuretics	Low-ceiling diuretics and potassium-sparing agents (C03EA)	0.9 (all), 1.4 (renal cancer)	Cox	Cox	None	None
TNF Inhibitor	Tumor necrosis factor alpha (TNF- ? ) inhibitors (L04AB)	0.53, 3.29 (all), 1.45 (Colorectal), 2 (NMSC)	None	None	None	None
Methotrexate	Nitrogen mustard analogues (L01AA)	1.6 (all), 2.8 (NMSC)	None	None	None	None
Predinisolone	Glucocorticoids (H02AB)	1.6 (all), 2.8 (NMSC)	Cox/CC	None	None	Cox

Drug descriptions are in first and second columns. Signal in Cox and/or CC indicates whether a tentative signal in Cox, case-crossover, or both analyses.
