# Supplementary material for: Systematic assessment of pharmaceutical prescriptions in association with cancer risk: a method to conduct a population-wide medication-wide longitudinal study
Source: Sci Rep. 2016 Aug 10;6:31308. doi: 10.1038/srep31308 (PMC4979093; doi:10.1038/srep31308)
Supplement: Supplementary Information [file srep31308-s1.pdf]

Supplementary Information

Systematic assessment of pharmaceutical prescriptions in association with cancer risk: a population-wide medication-wide longitudinal study

Chirag J Patel, Jianguang Ji, Jan Sundquist, John PA Ioannidis, Kristina Sundquist

Supplementary Table 1. Sample sizes for any, breast, colon, and prostate cancers in Sweden. Training and testing numbers are denoted in the table (separated by a /).

	**Any**	**Breast**	**Colon**	**Prostate**
2005	8223/8613	1301/1177	567/605	1901/1881
2006	20240/22684	2874/3016	14761564	4063/4512
2007	20134/22588	2790/3030	1447/1769	4094/4224
2008	20666/22680	2960/2983	1516/1667	3983/4159
2009	21909/23729	2909/3063	1499/1771	4812/4872
2010	21471/23225	3022/3284	1531/1693	4378/4456

Supplementary Figure Legends

Figure S1. Overall "Volcano" plot (-log10(p-value) vs. hazard ratio) of associations in any cancer, breast, colon, or prostate cancers, combining estimates from training and testing datasets for Cox analyses. Orange denotes tentative signals of association (p-value less than Bonferroni in both training and testing datasets). P-values lower than $1x10^{-100}$ set to $1x10^{-100}$ for clarity.

Figure S2. QQ-plot of p-values from Cox analyses. Test data shown in blue; training data shown in red). Deviation is seen from the unity line (solid black diagonal line) showing p-values generated from Cox analyses (y-axis) were lower than what is to be expected under the null hypothesis (x-axis).

Figure S3. Log(HR) of training versus testing data of Cox analyses. Orange color denotes a "tentative" signal (Bonferroni significance achieved in both the training and testing datasets) for each cancer type.

Figure S4. Cumulative density plot of effect sizes for Cox analyses. Orange line denotes distribution for tentative signals.

Figure S5. Overall "Volcano" plot (-log10(p-value) vs. odds ratio) of associations in any cancer, breast, colon, or prostate cancers combining estimates from training and testing datasets for case-crossover analyses. Orange denotes tentative signals of association (p-value less than Bonferroni in both training and testing datasets). P-values lower than $1x10^{-100}$ set to $1x10^{-100}$ for clarity.

Figure S6. QQ-plot for p-values from case-crossover analysis. Test data shown in blue; training data shown in red). Bonferroni threshold shown in solid horizontal black line. Deviation is seen from the unity line (solid black diagonal line) showing p-values generated from case-crossover analyses (y-axis) were lower than what is to be expected under the null hypothesis (x-axis).

Figure S7. Cumulative density plot of effect sizes for case-crossover analysis. Orange line denotes tentative signals.

Figure S8. Category-level and drug-level associations in any cancer. Tentative signals in the Cox regression are depicted in yellow. Highlighted are the C10 and C07 categories.

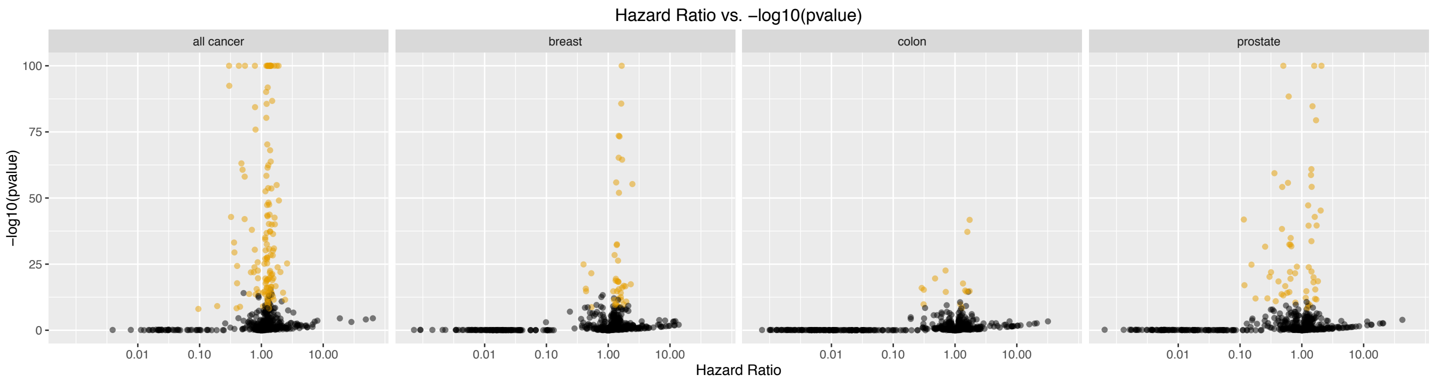

Figure S1. Overall "Volcano" plot (-log10(p-value) vs. hazard ratio) of associations in any cancer, breast, colon, or prostate cancers, combining estimates from training and testing datasets for Cox analyses. Orange denotes tentative signals of association (p-value less than Bonferroni in both training and testing datasets). P-values lower than $1x10^{-100}$ set to $1x10^{-100}$ for clarity.

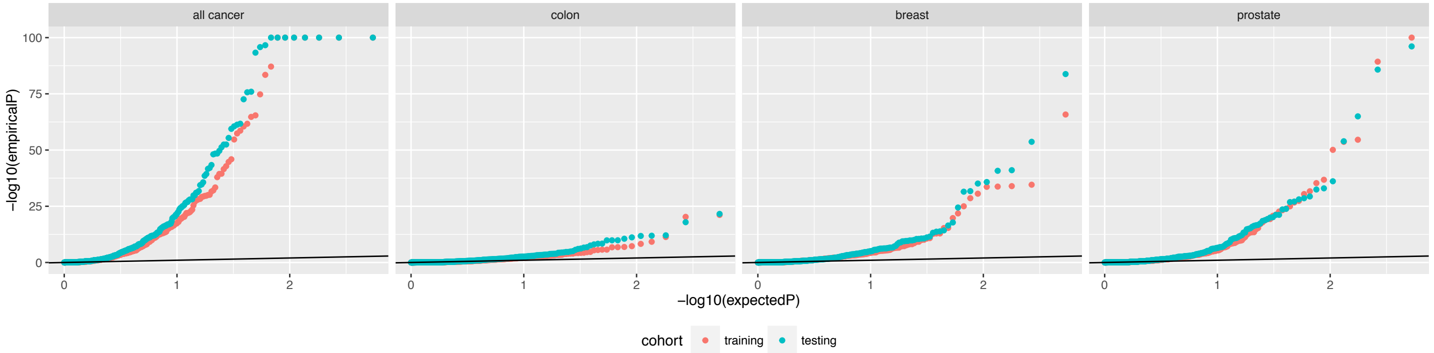

Figure S2. QQ-plot of p-values from Cox analyses. Test data shown in blue; training data shown in red). Deviation is seen from the unity line (solid black diagonal line) showing p-values generated from Cox analyses (y-axis) were lower than what is to be expected under the null hypothesis (x-axis).

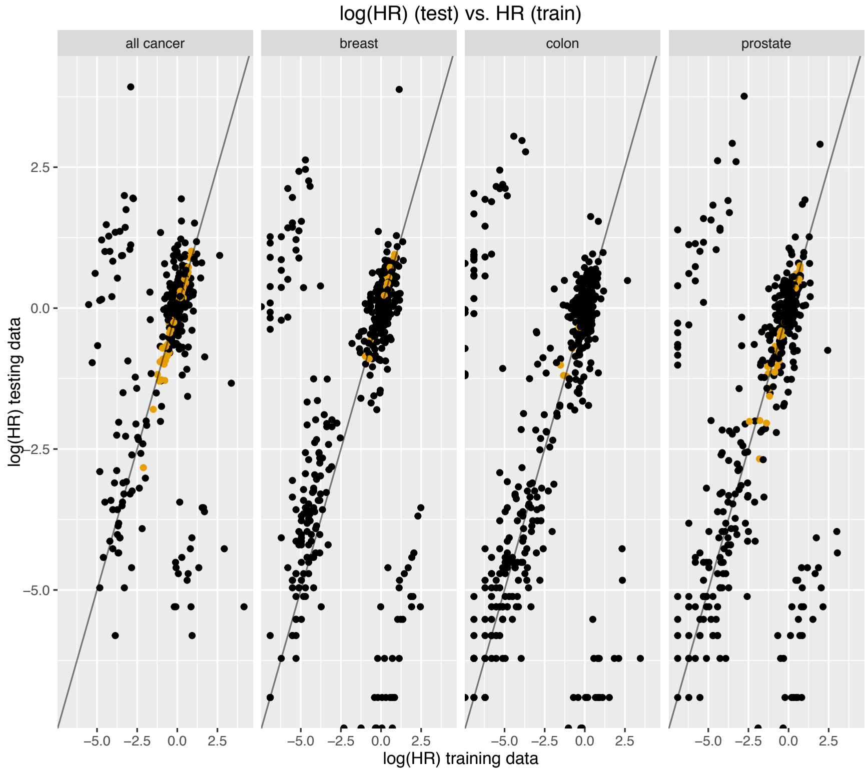

Figure S3. Log(HR) of training versus testing data of Cox analyses. Orange color denotes a "tentative" signal (Bonferroni significance achieved in both the training and testing datasets).

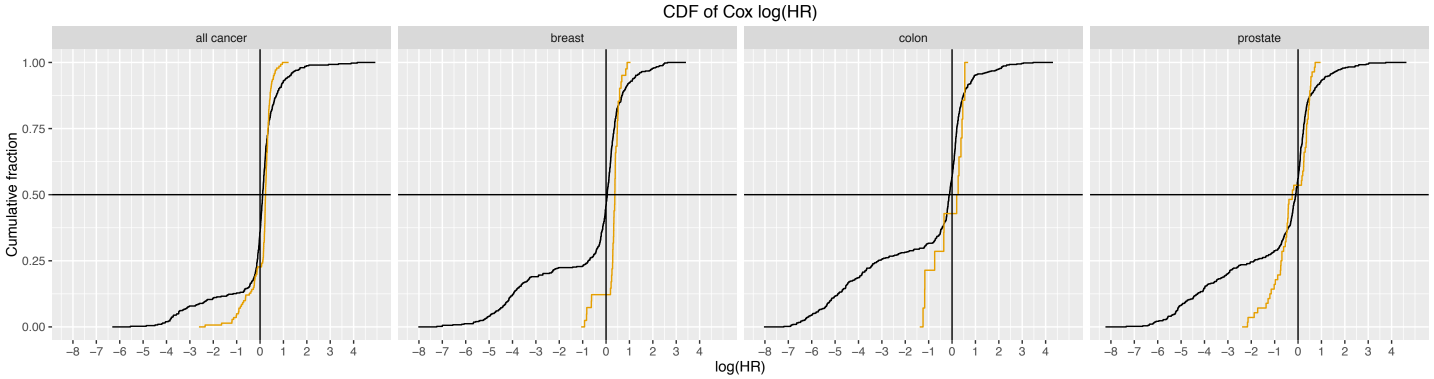

Figure S4. Cumulative density plot of effect sizes for Cox analyses. Orange line denotes distribution for tentative signals.

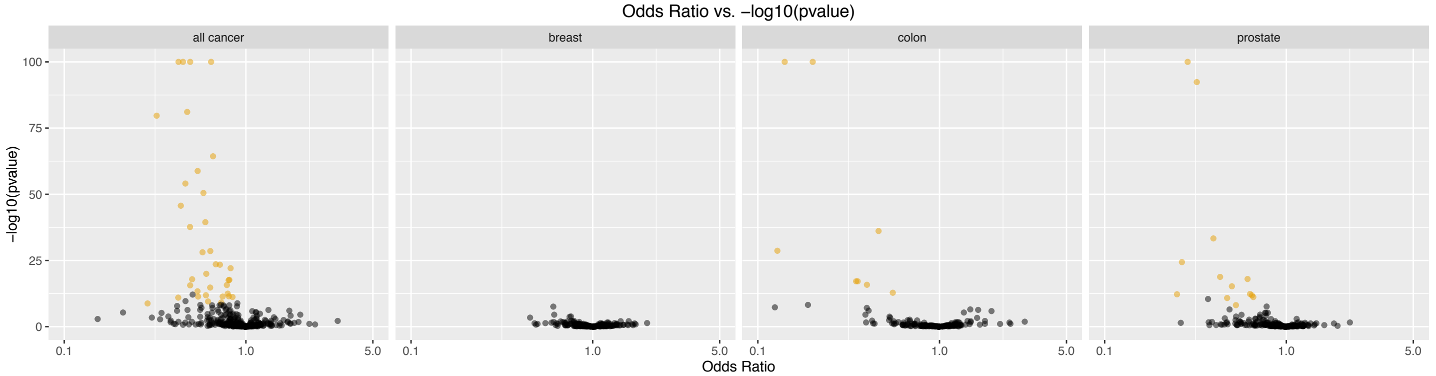

Figure S5. Overall "Volcano" plot (-log10(p-value) vs. odds ratio) of associations in any cancer, breast, colon, or prostate cancers combining estimates from training and testing datasets for case-crossover analyses. Orange denotes tentative signals of association (p-value less than Bonferroni in both training and testing datasets). P-values lower than $1x10^{-100}$ set to $1x10^{-100}$ for clarity.

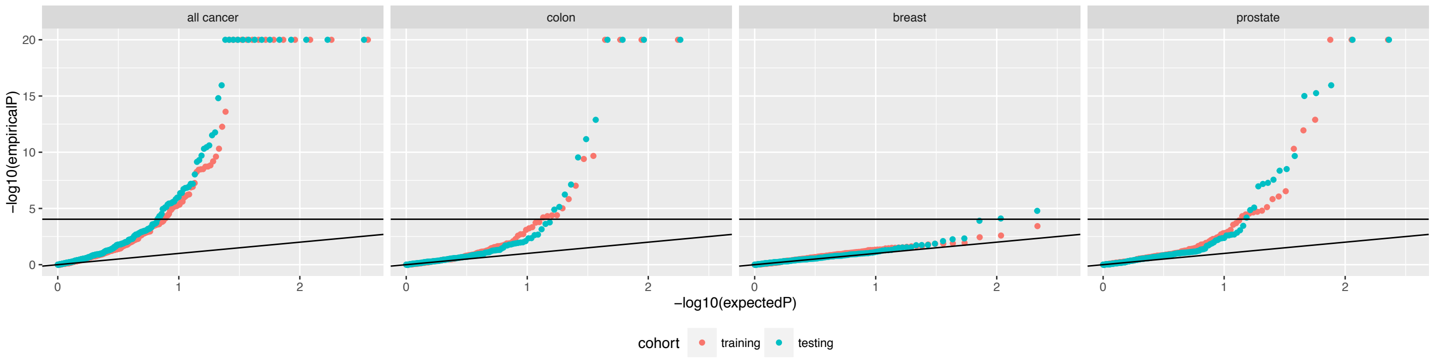

Figure S6. QQ-plot for p-values from case-crossover analysis. Test data shown in blue; training data shown in red). Bonferroni threshold shown in solid horizontal black line. Deviation is seen from the unity line (solid black diagonal line) showing p-values generated from case-crossover analyses (y-axis) were lower than what is to be expected under the null hypothesis (x-axis).

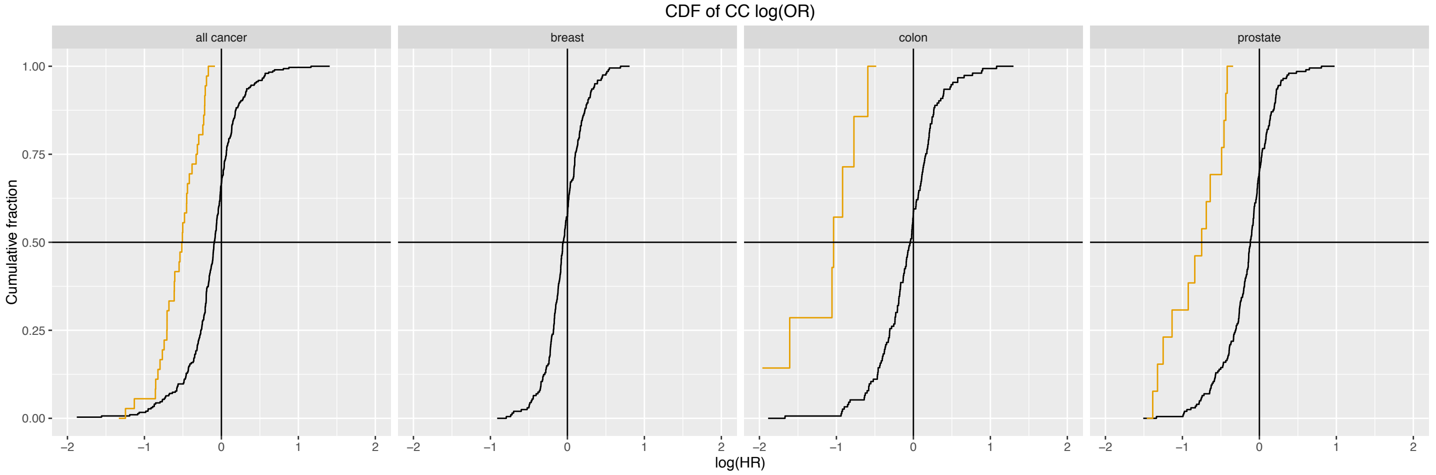

Figure S7. Cumulative density plot of effect sizes for case-crossover analyses. Orange line denotes tentative signals.

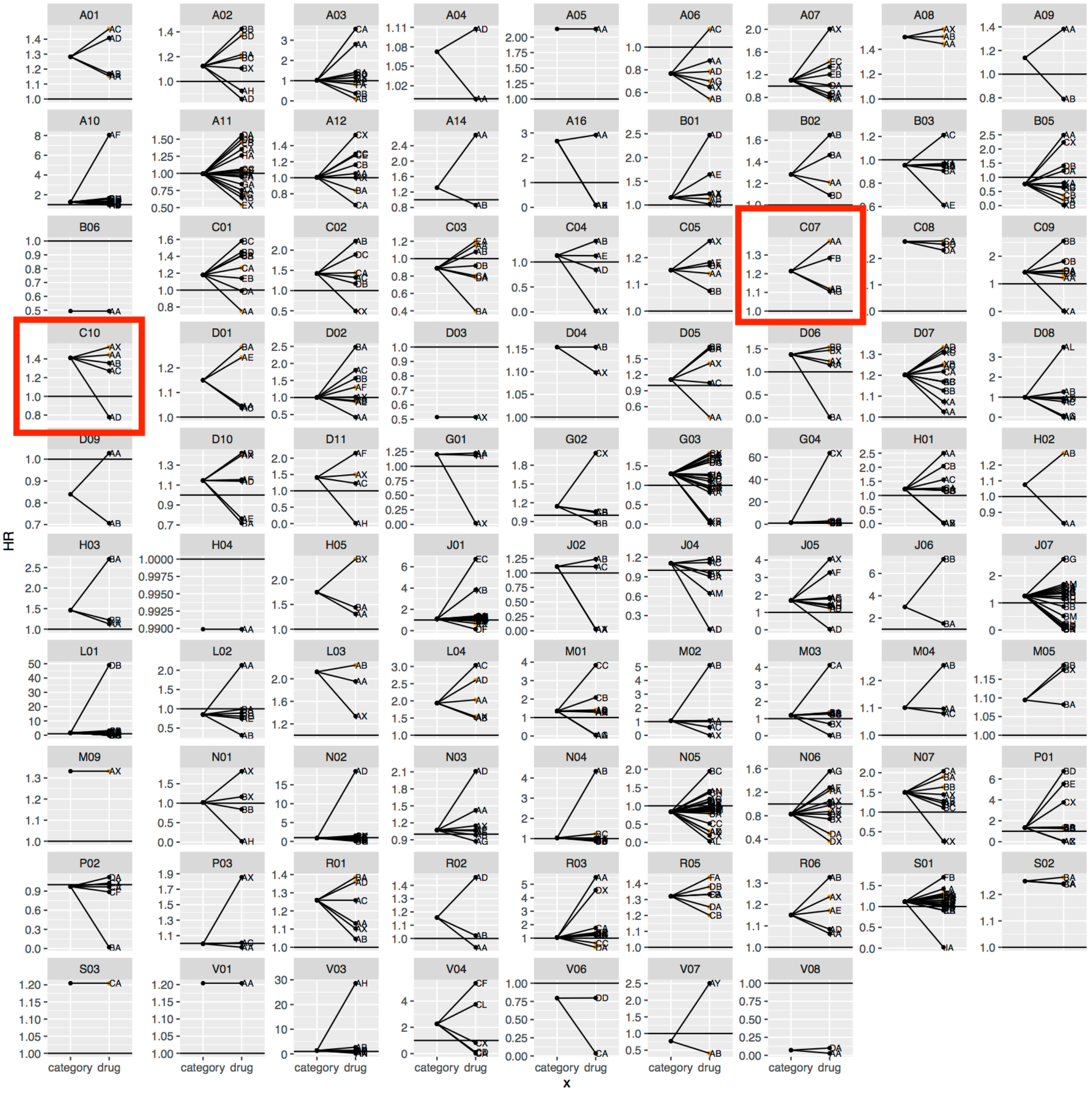

Figure S8. Category-level and drug-level associations in any cancer. Tentative signals in the Cox regression are depicted in yellow. Highlighted are the C10 and C07 categories.
